# Glycation marker glucosepane increases with the progression of osteoarthritis and correlates with morphological and functional changes of cartilage in vivo

**DOI:** 10.1186/s13075-018-1636-6

**Published:** 2018-06-22

**Authors:** Catherine Legrand, Usman Ahmed, Attia Anwar, Kashif Rajpoot, Sabah Pasha, Cécile Lambert, Rose K. Davidson, Ian M. Clark, Paul J. Thornalley, Yves Henrotin, Naila Rabbani

**Affiliations:** 1 Bone and Cartilage Research Unit, Arthropôle Liège, Institute of Pathology, Level 5, CHU Sart-Tilman, 4000 Liège, Belgium; 2 Warwick Systems Biology, University of Warwick, Clinical Sciences Research Laboratories, University Hospital, Coventry, CV2 2DX UK; 3 Warwick Medical School, Clinical Sciences Research Laboratories, University of Warwick, University Hospital, Coventry, CV2 2DX UK; 4 School of Computer Science, University of Birmingham, Birmingham, UK; 5 School of Biological Sciences, University of East Anglia, Norwich, UK; 6 Department of Physical Therapy and Rehabilitation, Princess Paola Hospital, Vivalia, Marche-en-Famenne, Belgium

**Keywords:** Glycation, Oxidative stress, Citrullination, Inflammation, Machine learning

## Abstract

**Background:**

Changes of serum concentrations of glycated, oxidized, and nitrated amino acids and hydroxyproline and anticyclic citrullinated peptide antibody status combined by machine learning techniques in algorithms have recently been found to provide improved diagnosis and typing of early-stage arthritis of the knee, including osteoarthritis (OA), in patients. The association of glycated, oxidized, and nitrated amino acids released from the joint with development and progression of knee OA is unknown. We studied this in an OA animal model as well as interleukin-1β-activated human chondrocytes in vitro and translated key findings to patients with OA.

**Methods:**

Sixty male 3-week-old Dunkin-Hartley guinea pigs were studied. Separate groups of 12 animals were killed at age 4, 12, 20, 28 and 36 weeks, and histological severity of knee OA was evaluated, and cartilage rheological properties were assessed. Human chondrocytes cultured in multilayers were treated for 10 days with interleukin-1β. Human patients with early and advanced OA and healthy controls were recruited, blood samples were collected, and serum or plasma was prepared. Serum, plasma, and culture medium were analyzed for glycated, oxidized, and nitrated amino acids.

**Results:**

Severity of OA increased progressively in guinea pigs with age. Glycated, oxidized, and nitrated amino acids were increased markedly at week 36, with glucosepane and dityrosine increasing progressively from weeks 20 and 28, respectively. Glucosepane correlated positively with OA histological severity (*r* = 0.58, *p* < 0.0001) and instantaneous modulus (*r* = 0.52–0.56; *p* < 0.0001), oxidation free adducts correlated positively with OA severity (*p* < 0.0009–0.0062), and hydroxyproline correlated positively with cartilage thickness (*p* < 0.0003–0.003). Interleukin-1β increased the release of glycated and nitrated amino acids from chondrocytes in vitro. In clinical translation, plasma glucosepane was increased 38% in early-stage OA (*p* < 0.05) and sixfold in patients with advanced OA (*p* < 0.001) compared with healthy controls.

**Conclusions:**

These studies further advance the prospective role of glycated, oxidized, and nitrated amino acids as serum biomarkers in diagnostic algorithms for early-stage detection of OA and other arthritic disease. Plasma glucosepane, reported here for the first time to our knowledge, may improve early-stage diagnosis and progression of clinical OA.

**Electronic supplementary material:**

The online version of this article (10.1186/s13075-018-1636-6) contains supplementary material, which is available to authorized users.

## Background

Osteoarthritis (OA) is a pathogenesis in movable joints characterized by cell stress and extracellular matrix degradation initiated by micro- and macroinjury. Pathogenesis involves low-grade inflammation mediated by innate and adaptive immunity and maladaptive repair responses. It initially manifests in molecular changes related to drivers of pathogenesis and culminates in anatomic and/or physiologic derangements of increasing severity [1].

Current diagnosis of OA is based on radiographic criteria and clinical symptoms, such as joint space width, pain, and loss of function. The related established cartilage lesions are irreversible with current treatments. Magnetic resonance imaging has been applied to early-stage OA diagnosis, but its use is limited by cost, availability, and absence of a validated scoring system. Measurement of soluble biomarkers in plasma or serum offers an alternative approach for early-stage diagnosis, assessment of progression, and therapeutic monitoring [2]. Development of early-stage diagnosis and effective earlier conservative interventions would likely decrease morbidity and cost of care.

**Fig. 1:**
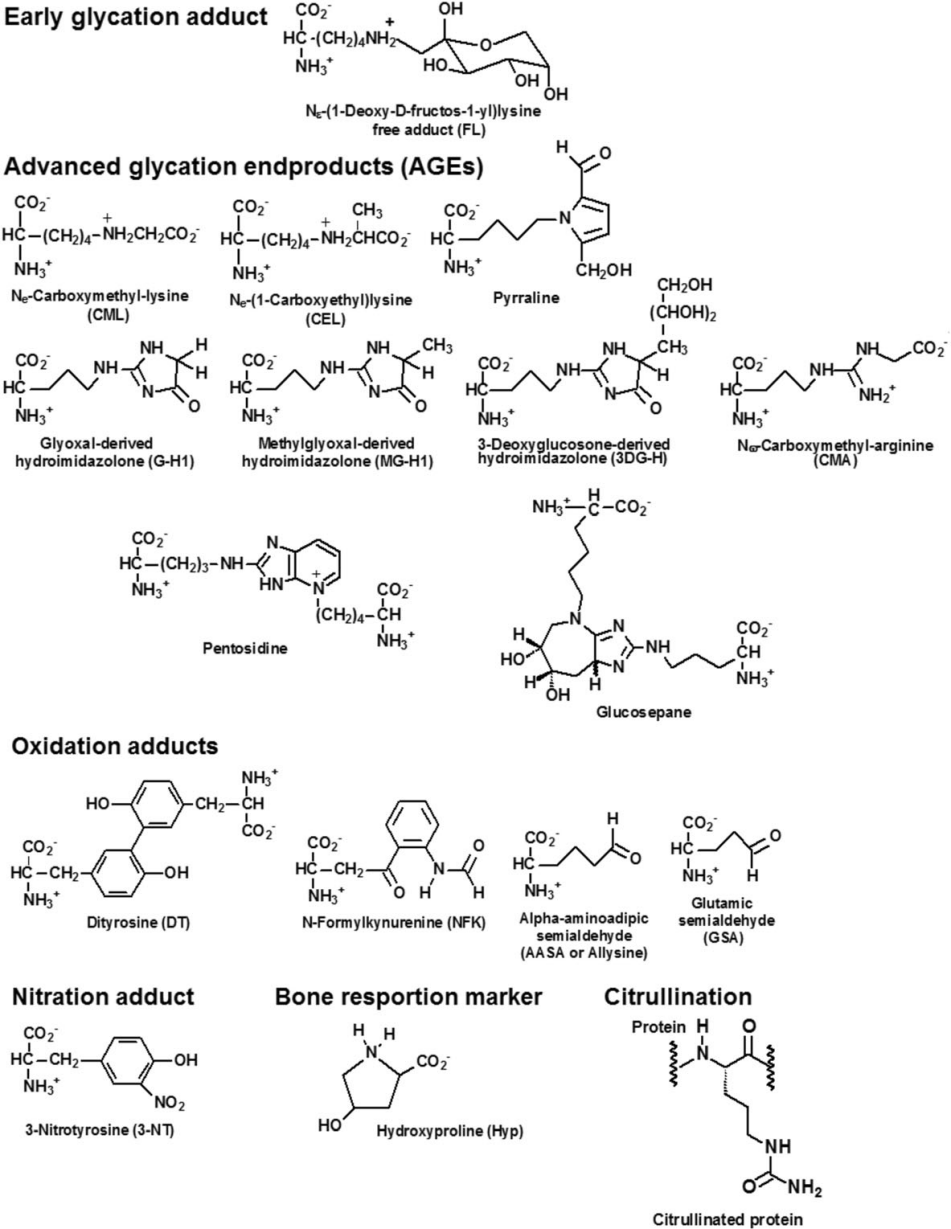
Protein glycation, oxidation, and nitration free adducts, bone resorption marker hydroxyproline, and citrullinated protein. For glycation, oxidation, and nitration adduct residues of proteins, the NH_3_^+^- and -CO_2_^−^ termini are part of the peptide backbone of the protein as -NH- and –CO- residues, respectively

Optimum candidate biomarkers in OA are molecules or molecular fragments present in cartilage, bone, or synovium [3]. Damaging posttranslational modifications of proteins in the joint—glycation, oxidation, and nitration—are considered part of the pathogenic process in OA, impairing biomechanical properties of cartilage [4]. A source of blood-based biomarkers relevant to damaging modifications of cartilage and other proteins are trace-level glycated, oxidized, and nitrated amino acids (Fig. 1), part of which originates from the arthritic joint. We recently reported patterns of changes of these metabolites in synovial fluid and plasma of patients with early and advanced stages of OA and other arthritic disease of the knee compared with subjects with good skeletal health. Patterns of analyte change were distinctive for type of arthritis. Data-driven combination of plasma or serum levels of glycated, oxidized, and nitrated amino acids with anticyclic citrullinated peptide (anti-CCP) antibody status and hydroxyproline (Hyp) by machine learning techniques with two different diagnostic algorithms applied sequentially provided high sensitivity and specificity for diagnosis and typing of early-stage arthritic disease [5]. The application of this diagnostic approach may be further substantiated by gaining insight into the temporal relationship of changes in glycated, oxidized, and nitrated amino acids in serum during development of experimental OA, where correlation with changes in joint histology and thickness and biomechanical properties of cartilage may be made. It is currently unknown how levels of serum glycated, oxidized, and nitrated proteins and amino acids change longitudinally in experimental models of OA and how the changes relate to morphological and functional changes of cartilage in the developing arthritic joint. Glucosepane (GSP) is a further major glycation-derived crosslink of joint proteins [6, 7] that we did not study in our previous work but was considered in the present study [5].

In this work, we studied the progression of histological and biomechanical properties in the Dunkin-Hartley guinea pig model of spontaneous OA. This animal model is the gold standard for studying aging-related OA, is recommended by the Osteoarthritis Research Society International (OARSI), and is defined as a spontaneous model of OA [8, 9]. It has advantages over other animal models in that the guinea pig knee joint structure is similar to that of the human knee and develops OA with many histopathological similarities to human pathology, related to both age and body weight [10]. The development of OA during a younger life period than in human subjects involves a shorter period for protein damage adduct accumulation than in human subjects, and hence changes in serum concentrations of glycated, oxidized, and nitrated amino acids may be smaller than those found in human subjects [5]. Nevertheless, serum GSP emerged in this animal model and in an in vitro model of chondrocyte inflammation as a key new glycated amino acid biomarker. This translated well to clinical early and advanced OA. This further advances the potential role of glycated, oxidized, and nitrated amino acids as biomarker features in diagnostic algorithms for early-stage detection and typing of OA and other arthritic disease.

## Methods

### Spontaneous OA in Dunkin-Hartley guinea pig

Sixty male 3-week-old Dunkin-Hartley guinea pigs, purchased from Charles River Laboratories (Paris, France) with identification by microchip, were used in the study. They were bred under pathogen-free conditions with free access to water. In experimental studies, they were housed three per solid-bottom cage and fed with a standard guinea pig chow (Special Diets Service, Essex, England) containing vitamin C (394 mg/kg) and vitamin D_3_ (1973 IU/kg), allowing 2 weeks for acclimatization. Polyvinyl chloride pipes were added to the cages to improve housing conditions and minimize stress. Separate groups of 12 animals were sacrificed and analyzed at age 4, 12, 20, 28 and 36 weeks. There was no repeated analysis of animals in the study. The number of animals per group was chosen according to the OARSI recommendation [11]. Animal body weight and food consumption were recorded weekly. Blood samples were collected by intracardiac puncture under general anesthesia (sodium pentobarbital 200 mg/kg intraperitoneally) immediately before animals were killed. Blood samples were centrifuged (2000 × *g*, 5 minutes), and serum was stored at − 80 °C until analysis. Samples were centrifuged within 1 hour of collection. All experimental procedures and protocols were reviewed and approved by the Institutional Animal Care and Use Ethics Committee of the University of Liège (Belgium) (reference 1648).

### Histology

At the time animals were killed, cartilage samples were processed for histological evaluation. The right knee joint (femoral condyles and tibial plateaus) from each animal was fixed for 24 hours in 4% paraformaldehyde, followed by decalcification in hydrochloric acid (DC2 medium; Labonord, Templemars, France) for 4 hours at 4 °C before embedding in paraffin. The right kidney and a piece of the liver were fixed in 4% paraformaldehyde and embedded in paraffin.

Sections (6 μm) of the femoral condyles and tibial plateaus were cut with a microtome in the central area not covered by meniscus following the Cushin plane, as recommended by OARSI [11]. Three sections at 200-μm intervals were stained with hematoxylin, Fast Green, and Safranin-O, and one supplementary central section was stained with toluidine blue. Each compartment of the section (tibial median, tibial lateral, femoral median, and femoral lateral) was scored by two trained experts blinded from sample identity following OARSI recommendations for the guinea pig model. Briefly, the evaluation considered the cartilage surface integrity (0–8), the proteoglycan content (0–6), the cellularity (0–3), the tidemark integrity (0–1), and the osteophyte (0–3), with a maximum of 21 per compartment. The mean score of three sections was calculated for each knee compartment. To assess the global OA score, scores of each compartment were added, giving a maximal score of 84. Lateral and medial synovial membranes were also scored (synovial lining cells hyperplasia 0–2, villous hyperplasia 0–3, degree of cellular infiltration by perivascular lymphocytes and mononuclear cells 0–5), and the mean of lateral and median membrane was calculated to assess the global synovial score (maximum score of 10) [11].

### Biomechanical testing by Mach-1® micromechanical tester

The left knee joint (femoral condyles and tibial plateaus) of each animal was used for testing the biomechanical properties of articular cartilage assessed using a Mach-1® micromechanical tester (Mach-1; Biomomentum Inc., Laval, QC, Canada) [12]. Prior to testing, samples were thawed at room temperature in PBS for 30 minutes to equilibrate before starting experiments. Subsequently, the femoral condyle or tibial plateau was fixed with LOCTITE® 4013 glue (Henkel, Stamford, CT, USA) in a small plastic container (Additional file 1: Figure S2). Throughout the testing, each sample was kept moist with PBS. Using top-view pictures of each sample, at least 50 positions per articular surface were tested using the automated indentation and thickness-mapping protocol. The instantaneous modulus—a measure of cartilage stiffness and cartilage thickness—was calculated using the Mach-1 analysis software (see Additional file 1).

### Primary culture of human chondrocytes

Human chondrocytes were cultured in multilayers in six-well plates and treated with interleukin-1β (IL-1β) [13]. Chondrocytes were isolated from human articular cartilage taken during the installation of total knee prosthesis. Cartilage samples were obtained from four adults (two men and two women) whose mean age was 70 years (range, 51–81 years). All specimens used were obtained with informed consent. This procedure was approved by the Ethics Committee of the Catholic University of Louvain (project no. B403201214793). Full-depth articular cartilage was excised and immersed in DMEM (with phenol red and 4.5 g/L glucose) supplemented with 4-(2-hydroxyethyl)-1-piperazineethanesulfonic acid (HEPES) 10 mM, penicillin 100 U/ml, and streptomycin 0.1 mg/ml (all from Lonza, Verviers, Belgium). After three washings, chondrocytes were released from cartilage by sequential enzymatic digestions with 0.5 mg/ml hyaluronidase type IV S (Sigma-Aldrich, Bornem, Belgium) for 30 minutes at 37 °C, 1 mg/ml pronase E (Merck, Leuven, Belgium) for 1 hour at 37 °C, and 0.5 mg/ml collagenase from *Clostridium histolyticum* type IA (Sigma-Aldrich) for 16 to 20 hours at 37 °C. The enzymatically isolated cells were then filtered through a nylon mesh (70 μm), washed three times, counted, and filled to the density of 0.25 × 10^6^ cells/ml of DMEM (with phenol red and 4.5 g/L glucose) supplemented with 10% FBS, 10 mM HEPES, 100 U/ml penicillin, 0.1 mg/ml streptomycin, 2 mM glutamine (all from Lonza), and 20 μg/ml proline (Sigma-Aldrich). After 21 days of culture, chondrocytes were treated in triplicate with recombinant human IL-1β (1.7 ng/ml; Roche Pharmaceuticals, Brussels, Belgium). The seeding density of the chondrocytes in the six-well plates was 50,000 cells/cm^2^. There was no passage of the cells; the cells overlap and form an extracellular matrix. Culture medium and IL-1β treatment were replaced at 3 and 6 days, and conditioned medium was removed at 3, 6, and 10 days and stored at − 20 °C until analysis.

### Patients, healthy subjects, and sampling

Patient recruitment, characteristics, and sampling were similar to those previously described [14]. Briefly, patients with early-stage OA (eOA) (*n* = 28), early-stage rheumatoid arthritis (eRA) (*n* = 35), and inflammatory joint disease other than rheumatoid arthritis (often self-resolving) (non-RA) (*n* = 32) were recruited. Criteria for eOA were subjects presenting with new-onset knee pain, normal radiographs of the symptomatic knee, and routine exploratory arthroscopy with macroscopic findings classified as grade I/II on the Outerbridge scale, and recruited at the Orthopaedic Clinics, University Hospital Coventry & Warwickshire (UHCW), Coventry, UK. Patients with eRA and non-RA were recruited within 5 months of the onset of symptoms of inflammatory arthritis at the Rapid Access Rheumatology Clinic, City Hospital, Birmingham, UK. Synovial fluid and peripheral venous blood samples were collected at initial presentation, and diagnostic outcomes were determined at follow-up. Diagnosis of eRA was made according to the 1987 American Rheumatism Association criteria [15]. Diagnosis of non-RA was made when alternative rheumatological diagnoses explained the inflammatory arthritis [16]. Criteria for these clinical classifications are similar to those suggested in consensus position statements and best practice statements [16, 17]. Healthy controls were recruited at participating clinical centers (*n* = 29) at UHCW. For healthy control subjects, inclusion criteria were no history of joint symptoms, arthritic disease, or other morbidity, and exclusion criteria were a history of injury or pain in either knee, taking medication (excepting oral contraceptives and vitamins), and any abnormality at physical examination of the knee.

Recruitment of patients with advanced OA (*n* = 38) immediately prior to total knee replacement (TKR) surgery (advanced osteoarthritis [aOA], pre-TKR) was done with written informed consent from patients referred for TKR to the Norfolk and Norwich University Hospitals NHS Trust (NNUH), Norwich, UK. Patients were screened for study eligibility criteria as described previously [14]. Eligible patients were males or postmenopausal females scheduled for TKR. This study was approved by the National Research Ethics Service Committee East of England, Cambridge South, UK (approval no. 2012ORTH06L [104-07-12]). All study procedures were performed in accordance with relevant laboratory guidelines and institutional regulations.

Peripheral venous blood samples were collected with ethylenediaminetetraacetic acid (EDTA) anticoagulant from healthy subjects and patients with eOA after overnight fasting. Venous blood samples for the eRA, non-RA, and aOA study groups were collected in the nonfasted state. For analytes studied, diurnal variation in plasma and serum was 13–25%, depending on the analyte, as described previously. Blood samples were centrifuged (2000 × *g*, 10 minutes), and the plasma and synovial fluid supernatant was removed and stored at − 80 °C until analysis. Samples were centrifuged within 1 hour of collection. Serum was available for eRA and non-RA study groups, and plasma was used for all others. Serum was comparable to plasma because nonprotein analytes were assessed. To confirm this, venous blood samples were collected with informed consent from human volunteers (*n* = 6; 4 female, 2 male; age 47.8 ± 15.8 years; BMI 25.9 ± 4.0 kg/m^2^). Ethical approval was given by East Midlands Regional Ethics Committee (reference 16/EM/0095). Serum and plasma (with EDTA anticoagulant) was prepared and assayed for the concentrations of glycated, oxidized, and nitrated amino acids as described below. There was no significant difference between analyte levels in serum and plasma by Wilcoxon signed-rank test.

### Analysis of glycated, oxidized, and nitrated protein and amino acids in serum/plasma

Glycation, oxidation, and nitration adduct residues and related precursor unmodified amino acid residues in plasma/serum protein were quantified in exhaustive enzymatic digests, with correction for autohydrolysis of hydrolytic enzymes [18, 19]. The concentrations of glycated, oxidized, and nitrated amino acids (free adducts) and hydroxyproline in plasma/serum were determined similarly in 10 kDa ultrafiltrate of plasma/serum and cell culture medium. Ultrafiltrate of plasma/serum (50 μl) was collected by microspin ultrafiltration (10 kDa cutoff) at 4 °C. Retained protein was diluted with water to 500 μl and washed in four cycles of concentration to 50 μl and dilution to 500 μl with water over the microspin ultrafilter at 4 °C. The final washed protein (100 μl) was delipidated and hydrolyzed enzymatically as described previously [19, 20]. Protein hydrolysate (25 μl, 32 μg equivalent) or ultrafiltrate (5 μl) was mixed with stable isotopic standard analytes (amounts as given previously) and analyzed by LC-MS/MS. Samples were analyzed using an ACQUITY™ ultra-high-performance liquid chromatography system with a Xevo-TQS LC-MS/MS mass spectrometer (Waters, Manchester, UK). Samples are maintained at 4 °C in the autosampler during batch analysis. The columns were 2.1 × 50-mm and 2.1 × 250-mm, 5-μm particle size Hypercarb™ (Thermo Fisher Scientific, Waltham, MA, USA) in series with programmed switching at 30 °C. Chromatographic retention was necessary to resolve oxidized analytes from their amino acid precursors to avoid interference from partial oxidation of the latter in the electrospray ionization source of the mass spectrometric detector. Analytes were detected by electrospray positive ionization and mass spectrometry multiple reaction monitoring (MRM) mode, where analyte detection response was specific for mass/charge ratio of the analyte molecular ion and major fragment ion generated by collision-induced dissociation in the mass spectrometer collision cell. The ionization source and desolvation gas temperatures were 120 °C and 350 °C, respectively; cone gas and desolvation gas flow rates were 99 and 900 L/h; and the capillary voltage was 0.60 kV. Argon gas (0.5 Pa) was in the collision cell. For MRM detection, molecular ion and fragment ion masses and collision energies optimized to ± 0.1 Da and ± 1 eV, respectively, were programmed [19]. In all sample analyses, the investigator was blinded from the sample identity. Analytes determined were as follows: glycation adducts *N*^ε^-fructosyl-lysine (FL), *N*^ε^-carboxymethyl-lysine (CML), *N*^ε^-carboxyethyl-lysine (CEL), *N*^ω^-carboxymethylarginine (CMA), glyoxal-derived hydroimidazolone (G-H1), methylglyoxal-derived hydroimidazolone (MG-H1), 3-deoxyglucosone-derived hydroimidazolone isomers (3DG-H), GSP, and pentosidine; oxidation adducts dityrosine (DT), *N*-formylkynurenine (NFK), α-aminoadipic semialdehyde (AASA), and glutamic semialdehyde (GSA); nitration adduct 3-nitrotyrosine (3-NT); and related amino acids [19] (*see* Fig. 1 for structures and expansion of acronyms). The biochemical and clinical significance is described elsewhere [6]. Protein adduct residues (normalized to their amino acid residue precursors; mmol/mol amino acid modified) and serum or plasma free adduct concentrations (μM or nM) are given. In culture medium, free adduct concentrations were corrected for cell number by normalizing to cellular DNA content.

### Citrullinated protein and hydroxyproline

Serum citrullinated protein (CP) and Hyp were analyzed by stable isotopic dilution analysis LC-MS/MS, as previously described [20].

### Machine learning

We developed algorithms using the clinical analyte data to distinguish the following four groups of subjects and patients: healthy control, eOA, eRA, and non-RA. The diagnostic algorithms were trained on the dataset using support vector machines [21]. The algorithm was validated by twofold cross-validation using five randomized repeat trials for improved robustness. A two-stage approach was taken: (1) to distinguish between disease and healthy control and (2) to distinguish between eOA, eRA, and non-RA. We used accuracy of case and control classification to optimize algorithm features. Diagnostic characteristics, including area under the ROC (AUROC), are given with 95% CI determined via bootstrap analysis. The contribution of each feature in the algorithms to classification accuracy was assessed by determining the change in AUROC when a feature was omitted from the algorithm and retrained; a negative change represents a valuable feature, and a positive change an adverse feature, for classification accuracy. Data were analyzed using MATLAB version R2017A software (MathWorks, Natick, MA, USA).

### Statistical analysis

Results are expressed as mean ± SEM unless otherwise stated. Following a normality test, one-way analysis of variance (ANOVA) with Tukey’s posttest was performed for histology, MACH-1, and amino acid analytes. Pearson’s correlations were performed between global OA score, parameters of MACH-1, and amino acid biomarkers. Given the asymmetric distribution of biomarkers, a logarithmic transformation was considered to satisfy the hypothesis of normality. ANOVA was applied to compare each biomarker between age groups. The same analysis was used to compare the different parameters between age groups. The association between the log-transformed biomarkers and the parameters was assessed by Pearson’s correlation. A multiple regression model (including as independent variables age, the parameter of interest, and an interaction term between these two factors) was constructed in order to investigate the influence of this parameter in the biomarker-age relationship (potential confounding factor). The results were considered to be significant at the 5% critical level (*p* < 0.05). There was no repeated analysis of the guinea pigs or human subjects, so repeated measures analysis is not applicable. For longitudinal analysis of multilayer cultures, to investigate a possible difference between the two groups, a mixed model with an undefined covariance matrix was applied to the data. The independent variables considered in this model were time, IL-1β treatment, and interaction between them. This statistical approach allowed us to compare biomarker production curves between the two groups while taking into account the presence of correlated data. For significance tests and correlation analysis of 14 glycated, oxidized, and nitrated amino acids and hydroxyproline analyzed in serum filtrate and 14 glycation, oxidation, and nitration adduct residues and CP in serum protein (15 analytes in each sample type), analyzed without preconceived hypothesis, a Bonferroni correction of 15 was applied. The predictive ability of these analytes for development of OA was studied by developing a partial least squares (PLS) regression model. The model was trained to learn to predict OA histological score from concentrations of serum glycated, oxidized, and nitrated amino acids (FL, CML, CEL, MG-H1, G-H1, 3DG-H, CMA, AASA, GSP, GSA, NFK, DT, 3-NT, pyrraline, Hyp, and CP) with the 4–36 weeks guinea pig study groups. Subsequent to training, the model was used to predict OA histological score for each guinea pig. The residual error between model predictions and the actual OA histological score was estimated as root mean squares error. Error at each individual stage and the overall error at all stages were estimated. Data analysis was performed with SAS version 9.4 for Windows statistical software (SAS Institute, Cary, NC, USA).

## Results

### Spontaneous OA in Dunkin-Hartley guinea pig

All 60 guinea pigs were examined daily during the study. Two guinea pigs died at study weeks 30 and 31. Guinea pig body weights at the time the animals were killed were (in grams, mean ± SD): week 4, 282 ± 9; week 12, 723 ± 54; week 20, 887 ± 61; weak 28, 978 ± 78; and week 36, 1016 ± 80. During the study, the five groups showed similar gains in body weight, with no difference observed between study groups of the same age. Food consumption declined progressively with guinea pig age (*see* Additional file 1). The liver and kidney were examined after animals were killed. No abnormalities were observed; the liver and adrenal gland weights were similar between guinea pigs of the same study group.

**Fig. 2:**
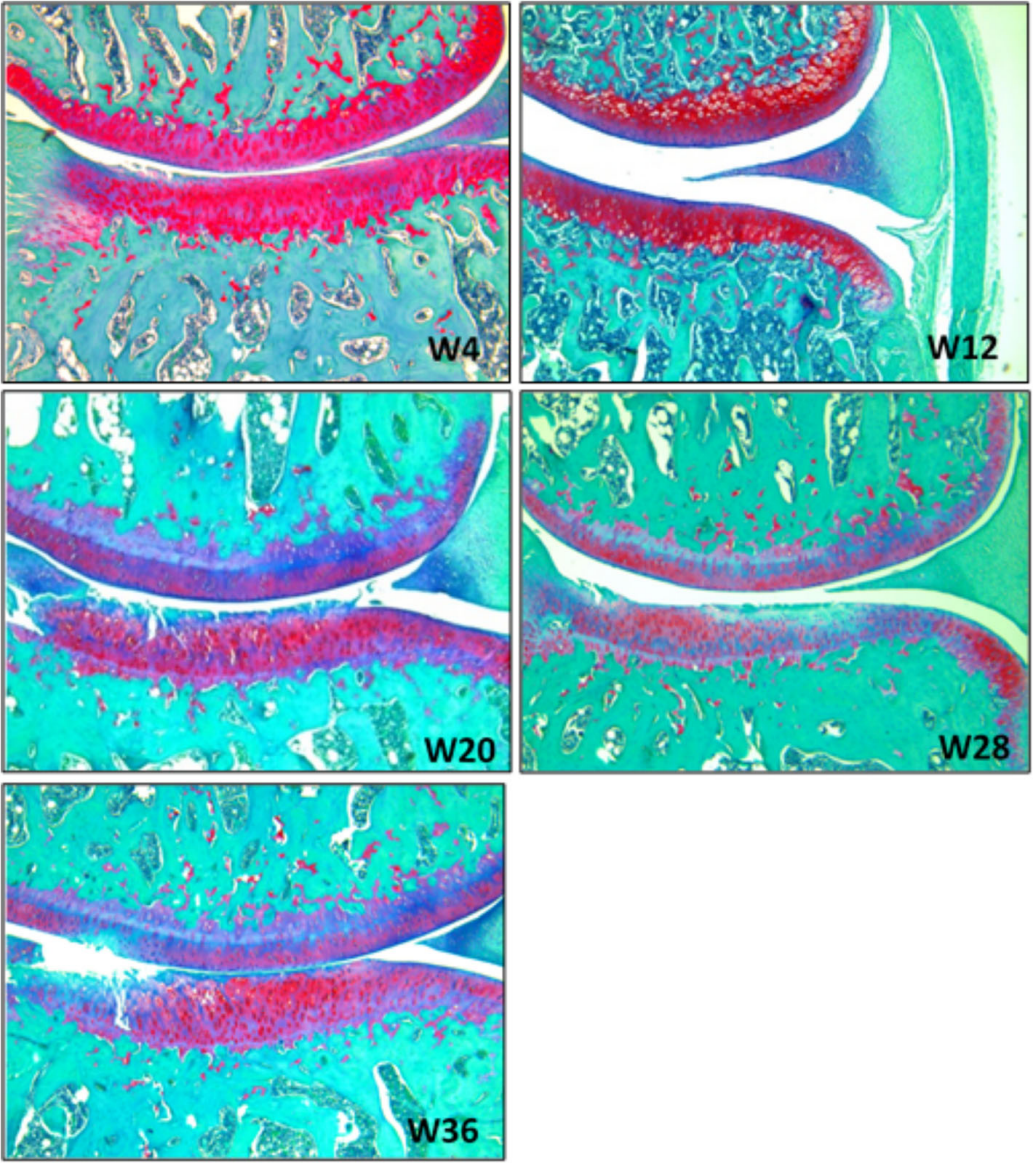
Representative pictures of medial compartment of right guinea pig knees of each group over time. Safranin-O/Fast Green/hematoxylin staining, 4 × magnification. W4–W36 (week 4 – week 6) inset indicates the age of the guinea pig donor analyzed

**Fig. 3:**
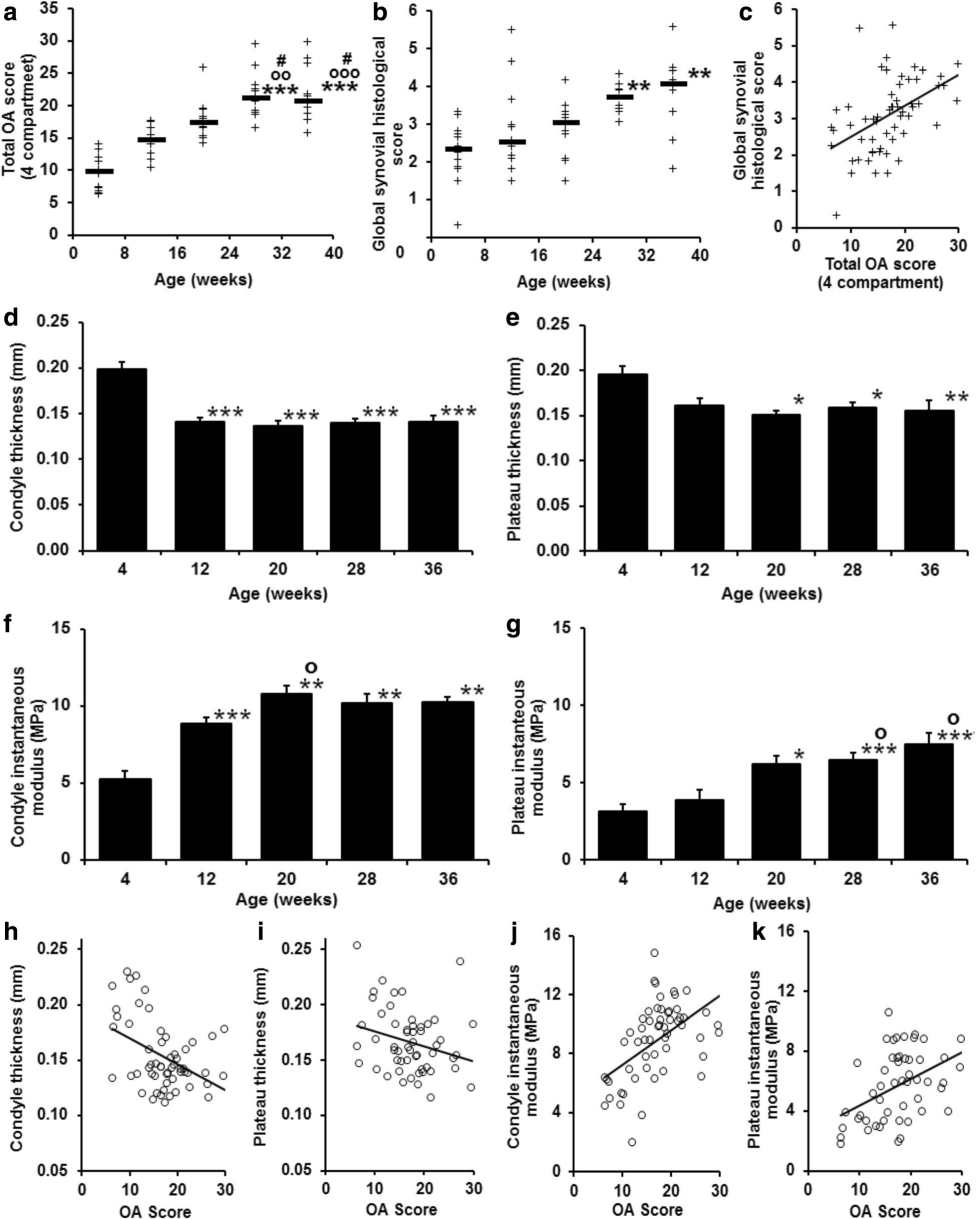
Development of osteoarthritis in Dunkin-Hartley guinea pigs. **a** Total OA score at four sites in each group. **b** Global synovial histological score in each group. Horizontal bars indicate median values. **c** Correlation of global synovial histological score with total OA score (*r* = 0.55, *p* = 7 × 10^− 6^; Spearman). Thickness (in millimeters) (**d** and **e**) and instantaneous modulus (MPa) (**f** and **g**) in femoral condyles and tibial plateau, respectively. Data are mean ± SEM (*n* = 12 in each group, except *n* = 10 at week 36). **h–k** Correlations of cartilage thickness and instantaneous modulus on global OA histological score in condyle and tibial plateau. Correlation coefficients are (**h**) *r* = − 0.35, *p* < 0.01; (**i**) *r* = − 0.27, *p* < 0.05; (**j**) *r* = 0.58, *p* < 0.001; and (**k**) *r* = 0.44, *p* < 0.002. Significance (**a–f**): * *p* < 0.05; ** *p* < 0.01; and *** *p* < 0.001 with respect to 4-week study group; o, oo, and ooo, *p* < 0.05, *p* < 0.01, and *p* < 0.001 with respect to 12-week study group; and # *p* < 0.05 with respect to 20-week study group; one-way analysis of variance with Tukey posttest

Histological assessment of cartilage lesions as recommended by OARSI showed that guinea pigs spontaneously developed severe knee OA (Fig. 2). In all animals, the global histological score increased significantly with age until week 28 and then stabilized between weeks 28 and 36 (Fig. 3a). A significant and progressive increase of synovial score between weeks 4 and 36 was observed (Fig. 3b). The global histological score correlated positively with the global synovial histological score (*r* = 0.55, *p* < 0.001).

Cartilage thickness and biomechanical properties were assessed by the Mach-1® micromechanical tester. Cartilage thickness of the knee joint was decreased at the condyle and tibial plateau regions from 12 to 20 weeks and remained at a stable low level thereafter (Fig. 3c and d). Instantaneous modulus of the articular cartilage increased progressively in the condyle at weeks 12 and 20 and remained at a stable high level thereafter, and it increased progressively from weeks 20 to 36 in the tibial plateau region (Fig. 3e and f). There were negative correlations of condyle and tibial plateau cartilage thickness with increased OA histological score and positive correlations with instantaneous modulus (Fig. 3g–j). Detailed histological analysis showed that the structure of the cartilage and the proteoglycan content correlated with the instantaneous modulus of the femoral condyle (*r* = 0.58, *p* < 0.001; *r* = 0.52, *p* < 0.001). At the tibial plateaus, the strongest associations were found between the cartilage structure and integrity of tidemark and the instantaneous modulus (*r* = 0.44, *p* < 0.002; *r* = 0.43, *p* < 0.002, respectively).

### Analysis of glycated, oxidized, and nitrated amino acids and protein in serum

**Fig. 4:**
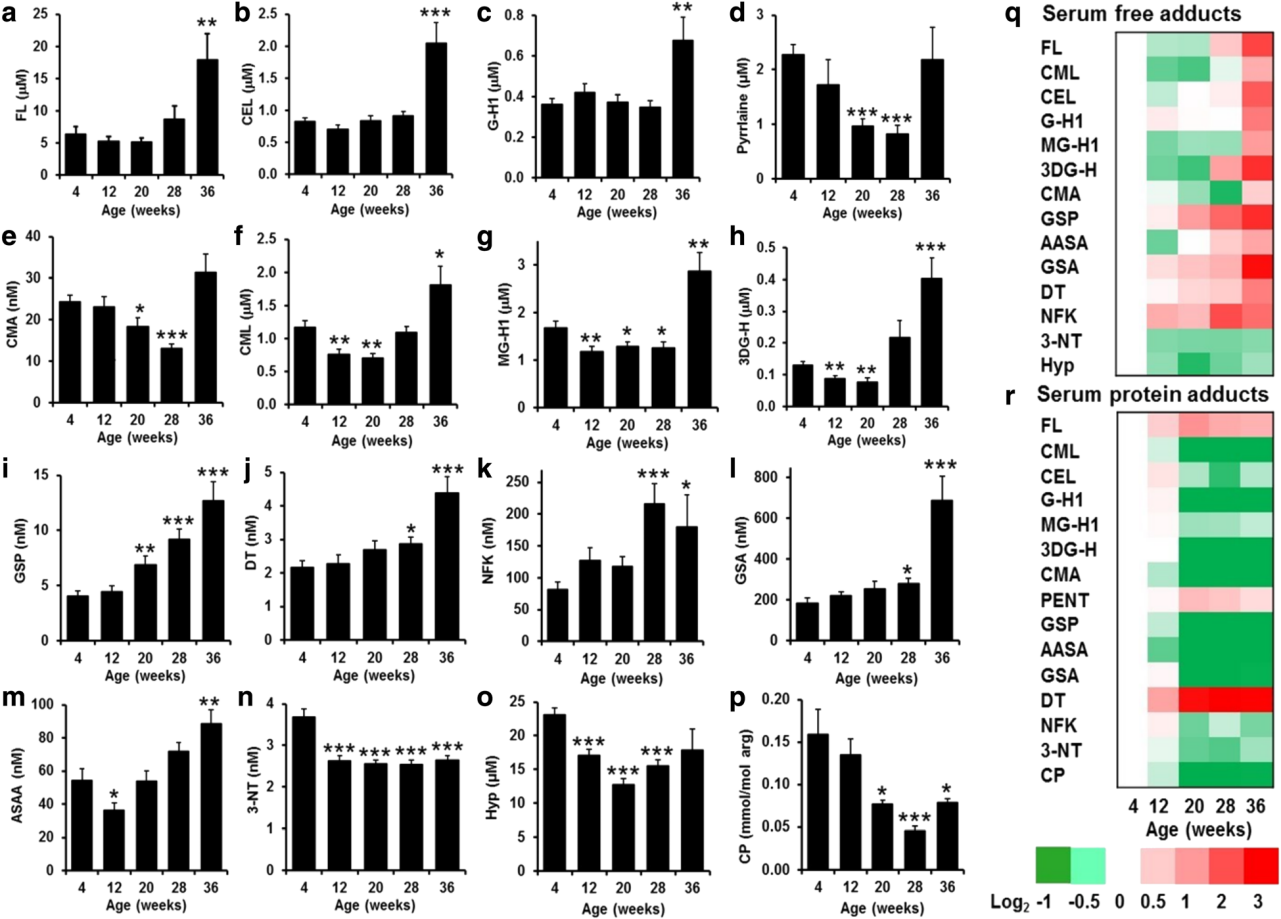
Serum protein glycation, oxidation, and nitration adducts and hydroxyproline and citrullinated protein during development of osteoarthritis in Dunkin-Hartley guinea pigs. Left side, center panels: Time course changes of serum glycation, oxidation, and nitration free adducts. **a** *N*^ε^-Fructosyl-lysine (FL). **b** *N*^ε^(1-carboxyethyl)lysine (CEL). **c** glyoxal-derived hydroimidazolone (G-H1). **d** Pyrraline. **e** *N*^ω^-carboxymethylarginine (CMA). **f** *N*^ε^(1-carboxymethyl)lysine (CML). **g** Methylglyoxal-derived hydroimidazolone (MG-H1). **h** 3-Deoxyglucosone-derived hydroimidazolone (3DG-H). **i** Glucosepane (GSP). **j** Dityrosine (DT). **k** *N*-formylkynurenine (NFK). **l** Glutamic semialdehyde (GSA). **m** α-Aminoadipic semialdehyde (AASA). (**n**) 3-Nitrotyrosine (3-NT). Other serum markers were Hydroxyproline (Hyp) (**o**) and citrullinated protein (CP) (**p**). Data are mean ± SEM. Significance: * *p* < 0.05, ** *p* < 0.01 and *** *p* < 0.001 by one-way analysis of variance with Tukey posttest. Right side panels: Heat map representation of changes: **q** serum glycation, oxidation, and nitration free adducts and Hyp. **r** Serum protein glycation, oxidation, and nitration adduct residues and CP

For glycated amino acids, serum concentrations of FL, CEL, and G-H1 were unchanged from 4 to 28 weeks and then increased two- to threefold at week 36 when OA was severe (Fig. 4a–c). Pyrraline free adduct was decreased at weeks 20 and 28 compared with 4, 12, and 36 weeks (Fig. 4d). Pyrraline is an advanced glycation endproduct (AGE) sourced exclusively from food, which may explain this disparate time-course profile [22]. CMA free adduct showed a similar trend (Fig. 4e). CML, MG-H1, and 3DG-H free adducts initially decreased at 12 and 20 weeks compared with the 4-week baseline levels, returned to baseline levels at 28 weeks, and then increased two- to threefold at 36 weeks (Fig. 4f–h). In contrast, GSP free adduct was unchanged at 12 weeks and then increased progressively from 20 to 36 weeks to threefold higher than baseline levels (Fig. 4i).

For oxidized amino acids, serum DT, NFK, and GSA free adducts increased progressively from 28 to 36 weeks to two- to threefold higher than baseline levels, increasing slightly later in OA development than GSP (Fig. 4j–l). AASA free adduct was decreased at 12 weeks and increased at 36 weeks (Fig. 4m). Serum 3-NT concentration was decreased by 29–32% at 12–36 weeks compared with baseline (Fig. 4n).

For OA-linked markers, bone resorption marker serum Hyp was decreased at weeks 12–28 with respect to baseline level, and serum CP was decreased by 50–71% from weeks 20 to 36 (Fig. 4o and p).

For glycation, oxidation, and nitration of serum protein, most adduct residue contents decreased from 12 to 20 weeks and remained decreased thereafter, exceptions being glycation adducts FL and pentosidine and oxidation adduct DT, which increased (*see* Additional file 1). Changes of serum free adducts and serum protein adducts are summarized in heat maps (Fig. 4q and r).

In correlation analysis, most serum glycation and oxidation free adducts were correlated with each other, the correlations being driven mainly by the marked increase of most analytes at 36 weeks. Exceptions were positive correlations of pyrraline with CMA and 3-NT free adducts and a negative correlation with NFK, and positive correlation of serum Hyp with CML. There were no correlations of glycation, oxidation, and nitration free adducts with serum CP. In contrast, serum CP correlated positively with levels of most glycation, oxidation, and nitration adduct residues of serum protein, except for FL and DT, where the correlations were negative (*see* Additional file 1).

For associations of protein glycation, oxidation, and nitration free adducts with the global histological score, GSP, AASA, GSA, DT, and NFK correlated positively with global histological score after correction for multiple analyte measurements. GSP had the highest correlation coefficient and significance (*r* = 0.58, *p* < 0.0001). In contrast, 3-NT free adduct correlated negatively with the global histological score. For cartilage thickness, 3-NT and Hyp correlated positively, and GSA negatively, at condyle and tibial plateau sites. Hyp had the highest correlation coefficient and significance (condyle *r* = 0.47, *p* = 0.0003; plateau *r* = 0.39, *p* = 0.003). Six free adducts correlated positively with instantaneous modulus; GSP had the highest correlation coefficient and significance (condyle *r* = 0.52 and plateau *r* = 0.56; *p* < 0.0001). CMA and 3-NT free adducts and CP correlated negatively with instantaneous modulus; 3-NT free adduct (condyle *r* = − 0.46, *p* = 0.0004; plateau *r* = − 0.41, *p* = 0.003) and CP (femoral condyle *r* = − 0.53, *p* < 0.0001), remaining significant after Bonferroni correction (Table 1).

**Table 1:** Correlation of glycation, oxidation, and nitration free adducts and citrullinated protein with global histological score and joint biomechanical properties measured by Mach-1 parameters

		Global histological score		Thickness				Instantaneous modulus			
				Condyle		Plateau		Condyle		Plateau	
		*r*	*p* Value	*r*	*p* Value	*r*	*p* Value	*r*	*p* Value	*r*	*p* Value
Glycation	FL	0.33	0.012							0.30	0.031
	G-H1	0.26	0.046								
	CMA							− 0.32	0.017		
	3DG-H	0.27	0.044								
	GSP	0.58	< 0.0001^a^					0.52	< 0.0001^a^	0.56	< 0.0001^a^
Oxidation	AASA	0.38	0.0029^a^					0.27	0.043	0.40	0.004
	GSA	0.36	0.0062^a^	− 0.29	0.033	− 0.33	0.015			0.35	0.013
	Dityrosine	0.42	0.0009^a^					0.34	0.010	0.36	0.010
	NFK	0.42	0.0011^a^					0.37	0.006	0.33	0.018
Nitration	3-NT	− 0.46	0.0003^a^	0.33	0.013	0.29	0.034	− 0.46	0.0004^a^	− 0.41	0.003^a^
Hyp				0.47	0.0003^a^	0.39	0.003^a^	− 0.38	0.0037		
CP		− 0.52	< 0.0001^a^					− 0.53	< 0.0001^a^	− 0.33	0.018

*Abbreviations: FL N*^ε^-fructosyl-lysine, *G-H1* Glyoxal-derived hydroimidazolone, *CMA N*^ω^-carboxymethylarginine, *3DG-H* 3-Deoxyglucosone-derived hydroimidazolone isomers, *GSP* Glucosepane, *AASA* α-Aminoadipic semialdehyde, *GSA* Glutamic semialdehyde, *NFK N*-formylkynurenine, *3-NT* 3-Nitrotyrosine, *Hyp* Hydroxyproline, *CP* Citrullinated protein

^a^Correlation coefficient significant after Bonferroni correction of 15 was applied

A PLS regression model of serum glycated, oxidized, and nitrated amino acids, Hyp, and CP on total OA histological score was computed. After training, the model was used to predict total OA histological score for each guinea pig. The outcome indicated that the model predicted histological score well in the early development of OA (4, 12, and 20 weeks) with declining predictive performance in more advanced stages (28 and 36 weeks) (*see* Additional file 1).

### Multilayer primary human chondrocytes culture

**Fig. 5:**
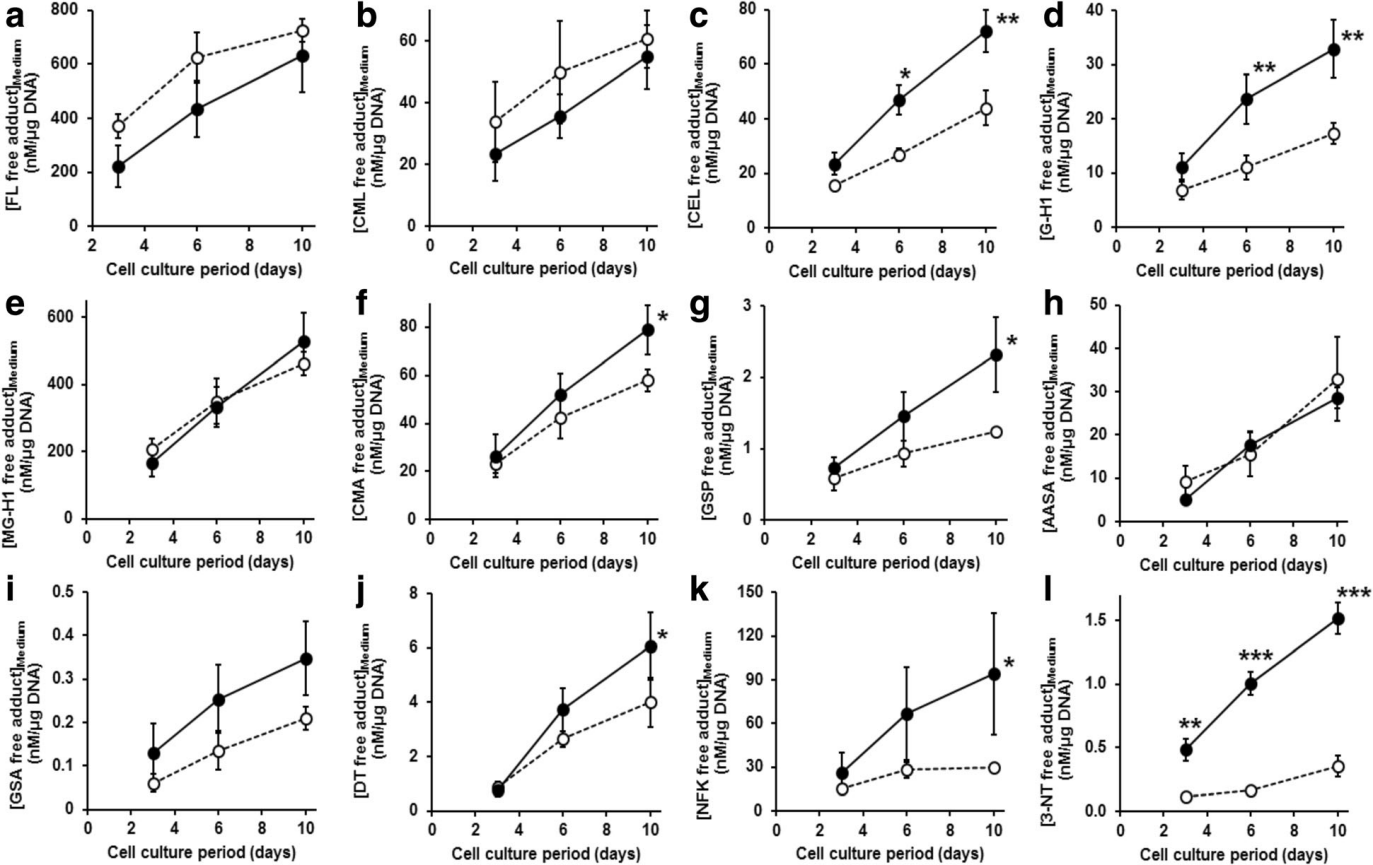
Concentrations of glycation, oxidation, and nitration free adducts in multilayer culture supernatant. Analyte concentrations in the culture medium, normalized to cell DNA content. Key: dashed lines = control; solid lines = + IL-1β. Free adduct: **a** FL. **b** CML. **c** CEL. **d** G-H1. **e** MG-H1. **f** CMA. **g** GSP. **h** AASA. **i** GSA. **j** DT. **k** NFK. **l** 3-NT. Data are mean ± SEM (*n* = 4). * *p* < 0.05, ** *p* < 0.01, and *** *p* < 0.001. Through longitudinal statistical study, we found that all biomarkers increased significantly over time (*p* < 0.0001). Comparison of the time-course curves showed that levels of G-H1, CEL, and 3-NT were increased with IL-1β treatment compared with control (*p* = 0.0023, *p* = 0.0071, and *p* < 0.0001, respectively)

From days 6 through 10, the production of CEL, G-H1, CMA, GSP, NFK, and 3-NT was increased with IL-1β treatment compared with control. In mixed model statistical study, all amino acid analytes increased over time, and increases of G-H1, CEL, and 3-NT were higher with IL-1β treatment compared with the control (Fig. 5).

### Plasma/serum glucosepane free adduct in clinical OA and application for clinical diagnosis and typing of early-stage arthritis of the knee

With the emergence of serum GSP free adduct as a potential marker of OA from the guinea pig study and chondrocyte studies, we analyzed serum GSP in patients and healthy controls, including a study group with aOA, pre-TKR surgery. Clinical characteristics were presented previously [14, 20]. Plasma GSP free adduct was increased 38% in eOA, sixfold in aOA, pre-TKR, twofold in non-RA, and threefold in eRA (Table 2). Developing diagnostic algorithms for early-stage arthritic disease, we found that optimum performance to discriminate between healthy controls and early-stage arthritis of any type was achieved with features in the algorithm: serum Hyp, glycation free adducts (CEL, GSP, MG-H1, 3DG-H, G-H1, and CMA), oxidation free adducts (NFK, methionine sulfoxide [MetSO], and DT), and nitration free adduct 3-NT (Algorithm 1). This had sensitivity 90.4%, specificity 83.2%, and AUROC 0.93; random selection is 0.50. In assessing the relative importance of each feature in classification accuracy, ΔAUROC on feature omission was (largest to smallest): CEL, − 0.067; GSP, − 0.063; Hyp, − 0.026; NFK, − 0.024; MetSO, − 0.014; MG-H1, − 0.011; 3DG-H, − 0.009; G-H1, − 0.008; 3-NT, − 0.004; DT, − 0.0005; and CMA, − 0.00008.

**Table 2:** Plasma or serum glucosepane free adduct in patients with early and advanced osteoarthritis and other early-stage arthritic disease

Study group	No.	Age (yr)	Gender (M/F)	Glucosepane (nM)
Control	29	34.4 ± 8.2	14/15	13.6 (10.1–18.1)
eOA	28	43.3 ± 13.3*	12/16	18.7 (13.3–35.5)*^,OOO^
aOA, pre-TKR	38	70.7 ± 8.9***	15/23	76.3 (61.2–97.5)***
Non-RA	32	51.7 ± 18.1**	14/16	31.2 (20.3–45.2)**^,OOO^
eRA	35	60.4 ± 15.7***	13/22	46.1 (31.1–77.8)***^,OOO^

*Abbreviations: eOA* Early-stage osteoarthritis, *aOA* Advanced osteoarthritis, *TKR* Total knee replacement, *RA* Rheumatoid arthritis, *eRA* Early-stage rheumatoid arthritis

Data are median (lower – upper quartile). Significance: 5-group comparison – *p* < 0.001 (*Kruskal-Wallis*). For binary comparisons: *, ** and ***, *p* < 0.05, *p* < 0.01 and *p* < 0.001 with respect to plasma levels of healthy controls; ooo, *p* < 0.001 with respect to plasma levels of aOA, pre-TKR (*Mann-Whitney U*)

In a subsequent step for patients with early-stage arthritis, optimum performance to discriminate between types of arthritis included features in the algorithm: anti-CCP-Ab positivity assessment with glycation free adducts (3DG-H, CML, FL, GSP, and CEL) and oxidation and nitration free adducts (MetSO and 3-NT, respectively). This distinguished eOA from eRA and non-RA with sensitivity 94.0%, specificity 96.1%, and AUROC 0.98; random selection is 0.33 (Table 3 and Additional file 1). In assessing relative importance of each feature in classification accuracy, mean ΔAUROC on feature omission for the three classifications was as follows: anti-CCP antibody status, − 0.116; 3DG-H, − 0.089; CML, − 0.082; FL, − 0.073; GSP, − 0.066; MetSO, − 0.034; CEL, − 0.027; and 3-NT, − 0.023.

**Table 3:** Characteristics of diagnostic algorithms for diagnosis and typing of early-stage arthritis: predictive algorithm outcomes for twofold cross-validation

Algorithm features	Algorithm 1: plasma Hyp and GSP, G-H1, MG-H1, 3DG-H, CEL, CMA, MetSO, 3-NT, NFK, and DT free adducts	Algorithm 2: anti-CCP-Ab positivity assessment and plasma GSP, FL, 3DG-H, CML, CEL, MetSO, and 3-NT free adducts		
Classification	Disease vs control	eOA vs non-RA and eRA	eRA vs non-RA and eOA	Non-RA vs eRA and eOA
Accuracy (%)	88.4 (86.9–90.0)	95.5 (93.7–97.3)	78.1 (74.3–82.0)	78.9 (74.7–83.2)
Sensitivity (%)	90.4 (88.7–92.1)	94.0 (88.8–99.3)	69.1 (54.4–83.7)	67.5 (46.2–88.8)
Specificity (%)	83.2 (77.4–89.0)	96.1 (92.6–99.6)	83.2 (71.8–94.6)	84.6 (77.1–92.1)
AUROC	0.93 (0.92–0.94)	0.98 (0.97–0.99)	0.86 (0.81–0.90)	0.88 (0.86–0.90)
Positive likelihood ratio	8.26 (5.77–10.75)	16.11 (9.56–22.66)	7.66 (2.94–12.37)	4.96 (3.31–6.60)
Negative likelihood ratio	0.11 (0.10–0.13)	0.06 (0.01–0.11)	0.34 (0.20–0.48)	0.36 (0.14–0.57)
Positive predictive value (%)	93.6 (91.7–95.6)	92.8 (86.5–99.0)	76.3 (65.1–87.4)	71.8 (62.7–80.8)
Negative predictive value (%)	77.3 (74.4–80.2)	97.5 (95.3–99.6)	84.4 (79.0–89.8)	86.2 (78.9–93.4)
F-score	0.92 (0.91–0.93)	0.93 (0.90–0.96)	0.69 (0.62–0.75)	0.64 (0.49–0.79)

*Abbreviations: MetSO* Methionine sulfoxide, *FL N*^ε^-fructosyl-lysine, *G-H1* Glyoxal-derived hydroimidazolone, *MG-H1* Methylglyoxal-derived hydroimidazolone, *CMA N*^ω^-carboxymethylarginine, *3DG-H* 3-Deoxyglucosone-derived hydroimidazolone isomers, *GSP* Glucosepane, *NFK N*-formylkynurenine, *3-NT* 3-Nitrotyrosine, *Hyp* Hydroxyproline, *eOA* Early-stage osteoarthritis, *TKR* Total knee replacement, *RA* Rheumatoid arthritis, *eRA* Early-stage rheumatoid arthritis, *DT* Dityrosine, *CCP* Cyclic citrullinated peptide, *CML N*^ε^-carboxymethyl-lysine, *CEL N*^ε^-carboxyethyl-lysine

Data are mean (95% CI). Analyte data other than GSP (Table 2) employed in algorithm development were reported previously [5]

## Discussion

In this study, we showed that serum concentrations of trace-level glycated, oxidized, and nitrated amino acids increase with development of OA in an experimental spontaneous model of knee joint OA. Multiple regression models, adjusted for body weight, suggested that these changes were not due to changes in body weight. GSP free adduct emerged as a biomarker that strongly and positively correlated with global OA histological score and instantaneous modulus measure of stiffness of articular cartilage, increasing with OA severity. Plasma GSP free adduct was modestly and markedly increased in early-stage and severe, advanced clinical OA, respectively. Inclusion of plasma GSP free adduct in a diagnostic algorithm with other trace-level glycated, oxidized, and nitrated amino acids improved detection and arthritis type classification of early-stage clinical OA. We also found that IL-1β, a key cytokine involved in OA pathogenesis, increased the release of GSP and other glycation, oxidation, and nitration free adduct release from chondrocytes, suggesting that inflammation-driven proteolysis may increase free adduct release in vivo. This provides further evidence that, taken together with our previous reports [5, 10, 20], suggests that measurement of trace-level damaged amino acids in serum or plasma are potential biomarkers for diagnosis, progression of severity and therapeutic monitoring in OA and other arthritic disease.

In advanced OA in guinea pigs at 36 weeks, serum concentrations of glycated and oxidized amino acids were increased compared with week 4 control, except for pyrraline, CMA, and 3-NT. From previous studies of Dunkin Hartley guinea pigs, the development of knee joint cartilage, proteoglycan, and bone structure is mature at 12 weeks [23]. Beyond this time, there is increased cartilage density associated with change in cartilage crosslink structure, and from 28 weeks, increased degradation of cartilage [24, 25]. Previous studies found increased markers of cartilage degradation, keratan sulfate, cartilage oligomeric matrix protein, and collagenase-generated fragments of collagen II [26] as well as markers of bone metabolism (urinary hydroxylysyl-pyridinoline and lysyl-pyridinoline, and serum osteocalcin), consistent with this [27]. We suggest that increases in glycated and oxidized amino acids are due to enhanced proteolysis of articular cartilage and bone remodeling, leading to increased flux of release of glycation and oxidation free adducts into the vasculature for urinary excretion. There is also an expected contribution from increased uptake of some glycated amino acids from food for some analytes (*see below*). Increased glycated and oxidized amino acid release from cartilage may occur without further change in its thickness through increased cartilage turnover and/or swelling [28]. The earlier increase in serum GSP from week 20 may relate to restructuring and decrease of cartilage crosslinks found during this period.

Pyrraline is an AGE derived only from food [22] and hence provides an objective biomarker of food consumption [29]. Serum CMA free adduct correlated positively with pyrraline and may be sourced mainly from the diet in this study. There was a progressive decline in food consumption by the guinea pigs from week 4 to week 36. Gait and mobility are impaired in advanced OA in this model [30], but in the present study decreased food consumption may have been linked to pain with loss of appetite and decreased voluntary activity. The guinea pigs typically show a progressive increase in body weight [31], as found in the present study (*see* Additional file 1). Serum pyrraline free adduct concentration showed a trend similar to that of food consumption with decreases at weeks 20 and 28, diverging from this with an anomalous increase at week 36. CML, MG-H1, and 3DG-H usually have significant contributions from the diet [29, 32, 33]. The decreases of serum CML, MG-H1, and 3DG-H free adduct concentrations at 12 weeks may be related to the initial decline in food consumption. Progressive decreased food consumption may explain decreases in serum CMA, CML, MG-H1, and 3DG-H free adduct concentrations at 20 weeks, as well as decreases in serum CMA and MG-H1 at 28 weeks. The lack of decrease in serum CML and 3DG-H free adduct concentrations at 28 weeks may relate to increasing release of these analytes from the joints with increasing OA progression. Reversal of the decrease in serum pyrraline concentration at 36 weeks is not linked to food consumption, but rather to increased efficiency of uptake of pyrraline from ingested food, likely mediated by increased intestinal amino acid transporter activity. Similar increased dietary uptake of CMA, CML, MG-H1, and 3DG-H is expected and may contribute to the increases of these serum analytes at 36 weeks. The mechanism by which this occurs merits further investigation.

The negative correlations of serum 3-NT free adduct with global histological score and condyle and plateau instantaneous modulus may be due to changes in 3-NT free adduct from digestion of nitrated proteins in the ingested chow and decreased food intake as OA developed because serum 3-NT free adduct correlated positively with serum pyrraline free adduct. A similar but more limited effect was found for serum CMA free adduct.

The amino acid analytes with strongest correlation to histological and biomechanical features of developing OA were GSP and DT free adducts. GSP is formed by degradation of FL residues and subsequent proteolysis of GSP-modified protein. Although GSP is present in food proteins, it is not usually absorbed from the diet. The strong link of serum GSP free adduct concentration to global OA histological score and cartilage stiffness is likely due to GSP being of an exclusive endogenous source, a major protein crosslink and formation by joint proteolysis. This also translated to increase plasma or serum levels of GSP in clinical OA. Serum DT free adduct may have emerged as a biomarker of global OA histological score for similar reasons and also its likely increased formation associated with inflammation.

We also measured protein glycation, oxidation, and nitration adduct residues and citrullination in serum protein during OA development. These showed markedly different changes with age (cf. Fig. 4q and r). This demonstrates the importance of analyzing protein glycation residues and free adduct separately. Most protein modifications were decreased as OA developed. Exceptions were FL, pentosidine, and DT. The concurrent decrease of many different modifications suggests the underlying cause may be increased capillary permeability with increased residence time of albumin in the interstitial fluid, where protein concentration and rates of protein modification are usually lower than in the vascular compartment [34]. Increased capillary permeability may be driven by increased inflammatory reaction from 3 to 12 weeks of age in this guinea pig model [35] and also by increased prostaglandin E_2_, a dilatator of blood vessels [10]. Levels of FL and pentosidine may be increased by decline in glucose tolerance related to insulin resistance driven by increased IL-1β [35], producing increased early-stage protein glycation and increased pentose-derived metabolite precursors of pentosidine [36]. The anomalous increase in DT while other oxidative markers are decreasing suggests a specific effect. Formation of DT occurs enzymatically by dual oxidase (DUOX) [37]. DUOX expression is increased through activation of activating transcription factor 2in inflammatory signaling [38]. Similar effects were found previously in plasma and synovial fluid protein in clinical early- and advanced-stage OA [5, 20]. This is a likely consequence of systemic low-grade inflammation in OA and indicates relevance of the Dunkin-Hartley guinea pig model of OA for clinical translation.

Serum CP decreased as OA developed in Dunkin-Hartley guinea pigs. The mechanism of this remains unclear, but it corroborates with our earlier clinical studies where there was higher plasma CP in patients with eOA than in patients with aOA [20]. The increased inflammatory mediators in OA are thought to reach their zenith in early-stage disease and then decrease in advanced disease [39]. Inflammatory mechanisms linked to CP formation through expression of protein arginine deiminases may be a component of this and may explain the decline of serum CP in advanced OA.

We studied the effect of IL-1β on flux of protein glycation, oxidation, and nitration, as judged by increased concentration of protein glycation, oxidation, and nitration free adducts in culture medium. IL-1β increased flux of formation of CEL, G-H1, CMA, GSP, DT, NFK, and 3-NT. This model was not confounded by change in uptake of protein glycation, oxidation, and nitration free adducts (cf. effects in Dunkin-Hartley guinea pigs). The upper limit of the concentration of IL-1β in human synovial fluid is about 20 pg/ml in patients with severe knee OA [40]. We used a higher concentration, as previously [41–43], to model the effects of continuous exposure to IL-1β in vivo at the steady-state concentration, with additions at 3-day intervals and half-life of 2.5 hours of IL-1β [44].

We applied plasma or serum glycated, oxidized, and nitrated amino acid with Hyp and anti-CCP antibody status for diagnosis and type of early-stage arthritic disease. For classification of good vs early-stage arthritic disease (any), the relative importance of algorithm features was as follows: CEL > GSP > Hyp > NFK > MetSO > MG-H1 > 3DG-H > G-H1 > 3-NT > DT > CMA. The high importance of GSP corroborates with the early changes in serum GSP free adduct found in experimental spontaneous OA in the present study, and the importance of Hyp corroborates results of our earlier studies [20]. The importance of methylglyoxal-derived AGEs, CEL, and MG-H1 is a new development and supports the emergence of the role of dicarbonyl stress in aging and chronic disease [45]. For classification of early-stage arthritic disease, the relative importance of algorithm features was as follows: anti-CCP antibody positivity > 3DG-H > CML > FL > GSP > MetSO > CEL > 3-NT. This reflects the high prevalence of anti-CCP antibody positivity in eRA. The three next most important features are glycation adducts, which may also suggest there are distinct contributions of glycation to eOA, eRA, and non-RA. Combination of estimates of serum trace-level glycated, oxidized, and nitrated amino acids and Hyp may therefore improve diagnosis of early-stage arthritic disease, including progression of experimental spontaneous OA, as supported by predictions with the PLS regression model.

## Conclusions

We conclude that trace-level damaged amino acids in serum may be valuable biomarkers in OA, particularly GSP.

## Additional file

Additional file 1: Supplementary text: description of measurement and outcome of guinea pig food consumption. **Figure S1.** (**a**) MACH-1 mechanical testing system. (**b**) View of a guinea pig femoral condyle with a position grid superimposed; **Figure S2.** Body weight of guinea pigs during the study. **Figure S3.** Partial least squares (PLS) regression model of serum glycated, oxidized, and nitrated amino acids Hyp and CP on total OA histological score. **Table S1.** Serum glycated, oxidized, nitrated, and citrullinated protein in the guinea pig model of osteoarthritis; **Table S2.** Correlation between glycation, oxidation, and nitration free adducts and hydroxyproline. **Table S3.** Correlations between glycated, oxidized, nitrated, and citrullinated serum protein. **Table S4.** Confusion matrix and nCorrect. (DOCX 735 kb)

## Abbreviations

3DG-H: 3-Deoxyglucosone-derived hydroimidazolone isomers
3-NT: 3-Nitrotyrosine
AASA: α-Aminoadipic semialdehyde
AGE: Advanced glycation endproduct
aOA: Advanced osteoarthritis
AUROC: Area under the receiver operating characteristic curve
CCP: Cyclic citrullinated peptide
CEL: *N*^ε^-carboxyethyl-lysine
CMA: *N*^ω^-carboxymethylarginine
CML: *N*^ε^-carboxymethyl-lysine
CP: Citrullinated protein
DT: Dityrosine
DUOX: Dual oxidase
EDTA: Ethylenediaminetetraacetic acid
eOA: Early-stage osteoarthritis
eRA: Early-stage rheumatoid arthritis
FL: *N*^ε^-fructosyl-lysine
G-H1: Glyoxal-derived hydroimidazolone
GSA: Glutamic semialdehyde
GSP: Glucosepane
HEPES: 4-(2-Hydroxyethyl)-1-piperazineethanesulfonic acid
Hyp: Hydroxyproline
IL-1β: Interleukin-1β
MetSO: Methionine sulfoxide
MG-H1: Methylglyoxal-derived hydroimidazolone
MRM: Multiple reaction monitoring
NFK: *N*-formylkynurenine
Non-RA: Inflammatory joint disease other than rheumatoid arthritis (often self-resolving)
OA: Osteoarthritis
OARSI: Osteoarthritis Research Society International
PLS: Partial least squares
TKR: Total knee replacement
