# Supplementary material for: Glycation marker glucosepane increases with the progression of osteoarthritis and correlates with morphological and functional changes of cartilage in vivo
Source: Arthritis Res Ther. 2018 Jun 22;20:131. doi: 10.1186/s13075-018-1636-6 (PMC6013878; doi:10.1186/s13075-018-1636-6)
Supplement: Supplementary file 1 — Supplementary text: description of measurement and outcome of guinea pig food consumption. Figure S1. (a) MACH-1 mechanical testing system. (b) View of a guinea pig femoral condyle with a position grid superimposed; Figure S2. Body weight of guinea pigs during the study. Figure S3. Partial least squares (PLS) regression model of serum glycated, oxidized, and nitrated amino acids Hyp and CP on total OA histological score. Table S1. Serum glycated, oxidized, nitrated, and citrullinated protein in the guinea pig model of osteoarthritis; Table S2. Correlation between glycation, oxidation, and nitration free adducts and hydroxyproline. Table S3. Correlations between glycated, oxidized, nitrated, and citrullinated serum protein. Table S4. Confusion matrix and nCorrect. (DOCX 735 kb) [file 13075_2018_1636_MOESM1_ESM.docx]

**Additional file 1**

**Supplementary text**

**Guinea pig food consumption**

Food, pelleted chow, was given *ad libitum*. Food intake was measured as decrease of chow weight in cage feed hoppers after a 24 h period, with correction of chow found in the cage litter. Food consumption of the guinea pigs from weeks 8 - 36 was (mean ± SD): 65 ± 3, 57 ± 4, 49 ± 2, 43 ± 4 and 43 g ± 2 per 24 h. There was, therefore, a steady decline in food consumption with age in the study group guinea-pigs from weeks 8 - 28, concomitant with a progressive slowing of increase in body weight.

**Supplementary figures**

**B.**

**A.**

**A**


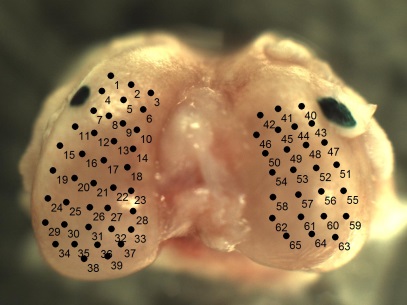


**Figure S1** MACH-1 mechanical testing system (Biomomentum, Canada). (B) View of a guinea pig femoral condyle with a position grid superimposed.


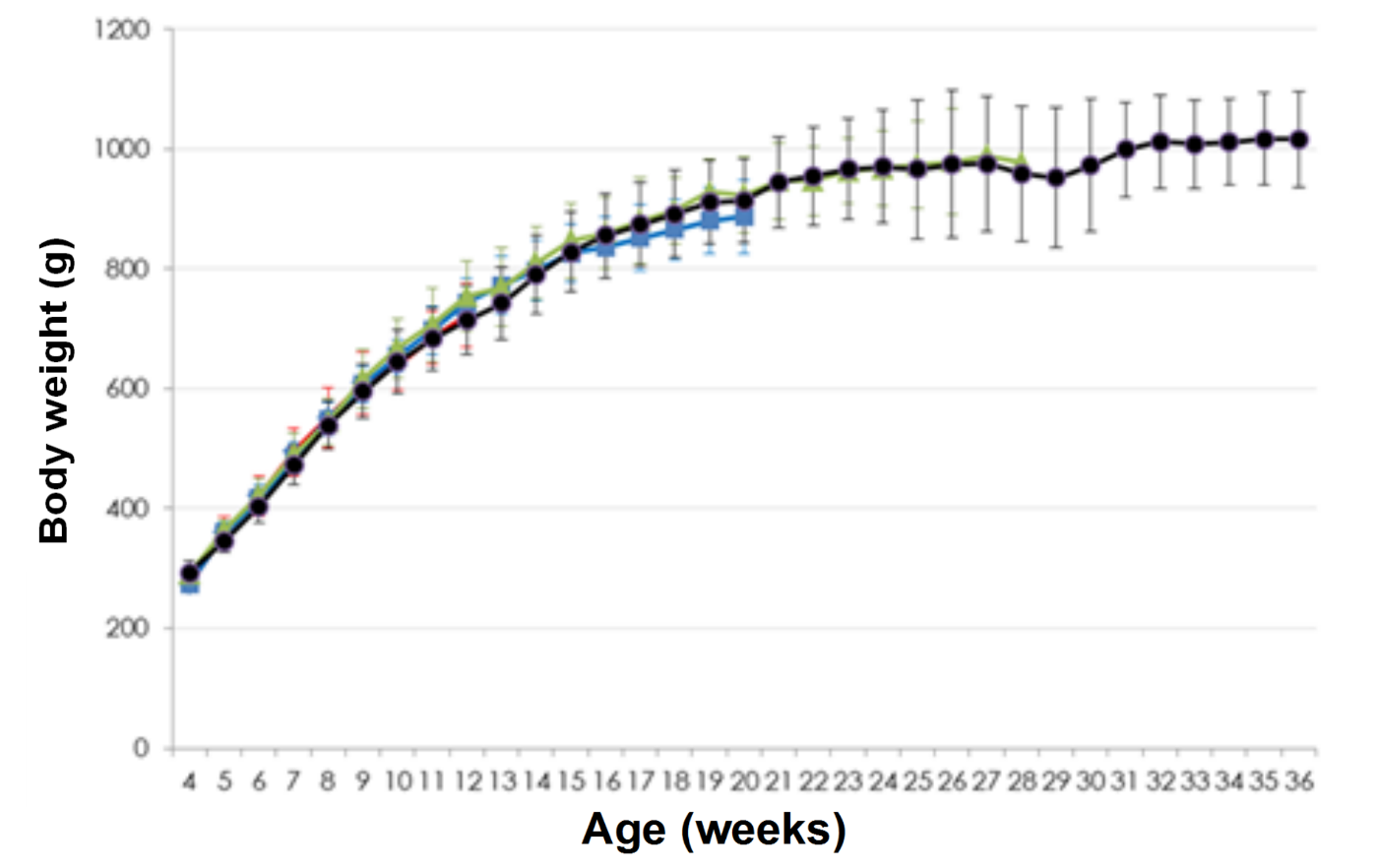
**Figure S2** Body weight of Dunkin Hartley guinea pigs in the study. Key (colour code, week of sacrifice): ●▬●, 12; ■▬■, 20; ▲▬▲, 28; and ●▬●, 36. Data are mean ± SD (n = 12 except for week 30, n = 11, and from week 31, n = 10).


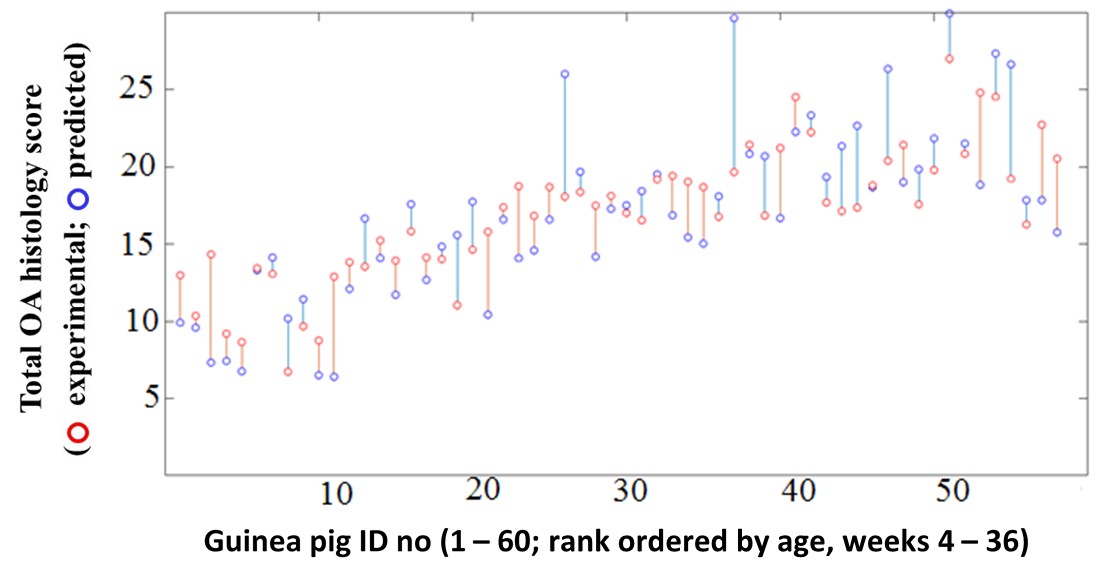


**Figure S3.** Partial least squares (PLS) regression model of serum glycated, oxidised and nitrated amino acids, Hyp and CP on Total OA histology score. The residual error between model predictions and the actual OA histology score was estimated as root mean squares error (RMSE). Error at each individual stage and the overall error at all stages was estimated. Mean RMSE by guinea pig age (weeks) was: 4, 3.32; 12, 3.00; 20, 3.16; 28, 4.39; 36, 4.07; and average (all) 3.61.

**SUPPLEMENTARY TABLES**

**Table S1** Serum glycated, oxidized, nitrated and citrullinated protein in the guinea pig model of osteoarthritis.

|  | Age (weeks) | | | | |
| --- | --- | --- | --- | --- | --- |
| Analyte | 4 | 12 | 20 | 28 | 36 |
| FL (mmol/mol lys) | 2.82 ± 0.78 | 3.70 ± 1.51 | 5.16 ± 0.84*** | 4.52 ± 0.99*** | 4.34 ± 0.90*** |
| CML (mmol/mol lys) | 0.564 ± 0.187 | 0.481 ± 0.211 | 0.206 ± 0.048*** | 0.183 ± 0.026*** | 0.218 ± 0.030*** |
| CEL (mmol/mol lys) | 0.090 ± 0.068 | 0.090 ± 0.061 | 0.093 ± 0.038 | 0.066 ± 0.030 | 0.095 ± 0.034 |
| G-H1 (mmol/mol arg) | 0.321 ± 0.285 | 0.333 ± 0.279 | 0.017 ± 0.006** | 0.018 ± 0.009** | 0.018 ± 0.007** |
| MG-H1 (mmol/mol arg) | 0.246 ± 0.199 | 0.270 ± 0.144 | 0.202 ± 0.042 | 0.203 ± 0.089 | 0.221 ± 0.029 |
| 3DG-H (mmol/mol arg) | 0.263 ± 0.117 | 0.296 ± 0.198 | 0.099 ± 0.057*** | 0.061 ± 0.023*** | 0.054 ± 0.016*** |
| CMA (mmol/mol arg) | 0.524 ± 0.255 | 0.416 ± 0.314 | 0.034 ± 0.019*** | 0.050 ± 0.015*** | 0.076 ± 0.016*** |
| Pentosidine (mmol/mol lys) | 0.0014 ± 0.0004 | 0.0015 ± 0.0008 | 0.0021 ± 0.0005** | 0.0020 ± 0.0002*** | 0.0018 ± 0.0005* |
| GSP (mmol/mol lys) | 0.175 ± 0.255 | 0.163 ± 0.126 | 0.093 ± 0.026 | 0.078 ± 0.026 | 0.095 ± 0.036 |
| AASA (mmol/mol lys) | 1.24 ± 0.35 | 0.79 ± 0.30** | 0.50 ± 0.16*** | 0.48 ± 0.15*** | 0.55 ± 0.24*** |
| GSA (mmol/mol arg) | 0.283 ± 0.223 | 0.236 ± 0.103 | 0.108 ± 0.030* | 0.115 ± 0.018* | 0.141 ± 0.029 |
| DT (mmol/mol tyr) | 0.034 ± 0.044 | 0.077 ± 0.074 | 0.174 ± 0.069*** | 0.190 ± 0.060*** | 0.185 ± 0.111*** |
| NFK(mmol/mol trp) | 0.251 ± 0.158 | 0.265 ± 0.137 | 0.163 ± 0.035 | 0.207 ± 0.031 | 0.168± 0.048 |
| 3-NT (mmol/mol tyr) | 0.0104 ± 0.006 | 0.0097 ± 0.0023 | 0.0099 ± 0.0027 | 0.0095 ± 0.0039 | 0.0117 ± 0.0038 |

|  |
| --- |

**Table S2** Correlation between glycation, oxidation and nitration free adducts and hydroxyproline.

|  | Glycation, oxidation and nitration free adduct and Hyp | FL |  |  |  |  |  |  |  |  |  |  |  |  |  |  |  |
| --- | --- | --- | --- | --- | --- | --- | --- | --- | --- | --- | --- | --- | --- | --- | --- | --- | --- |
|  |  | CML |  | 0.69 |  |  |  |  |  |  |  |  |  |  |  |  |  |
|  |  | CEL |  | 0.69 | 0.88 |  |  |  |  |  |  |  |  |  |  |  |  |
|  |  | G-H1 |  | 0.66 | 0.86 | 0.92 |  |  |  |  |  |  |  |  |  |  |  |
|  |  | MG-H1 |  | 0.77 | 0.85 | 0.91 | 0.84 |  |  |  |  |  |  |  |  |  |  |
|  |  | 3DG-H |  | 0.83 | 0.72 | 0.79 | 0.69 | 0.85 |  |  |  |  |  |  |  |  |  |
|  |  | CMA | 0.73 |  |  |  |  | 0.44 |  |  |  |  |  |  |  |  |  |
|  |  | GSP |  | 0.51 | 0.60 | 0.60 | 0.50 | 0.52 | 0.59 |  |  |  |  |  |  |  |  |
|  |  | AASA |  | 0.56 | 0.57 | 0.56 | 0.50 | 0.60 | 0.64 |  | 0.47 |  |  |  |  |  |  |
|  |  | GSA |  | 0.73 | 0.63 | 0.70 | 0.69 | 0.66 | 0.65 | 0.52 | 0.49 | 0.62 |  |  |  |  |  |
|  |  | DT |  | 0.48 | 0.65 | 0.77 | 0.74 | 0.75 | 0.63 |  | 0.59 | 0.54 | 0.61 |  |  |  |  |
|  |  | NFK | - 0.47 |  |  |  |  |  |  | - 0.43 | 0.47 |  |  |  |  |  |  |
|  |  | 3-NT | 0.52 |  |  |  |  |  |  |  |  |  |  |  | - 0.43 |  |  |
|  |  | Hyp |  |  | 0.59 |  |  |  |  |  |  |  |  |  |  |  |  |
|  |  |  | Pyrraline | FL | CML | CEL | G-H1 | MG-H1 | 3DG-H | CMA | GSP | AASA | GSA | DT | NFK | 3-NT | Hyp |
|  |  |  | Glycation, oxidation and nitration free adduct and Hyp | | | | | | | | | | | | | | |

Correlations statically significance (P<0.05) after a Bonferroni correction of 15 was applied.

**Table S3** Correlations between glycated, oxidized, nitrated and citrullinated serum protein.

|  | Serum adduct residue | CML | - 0.64 |  |  |  |  |  |  |  |  |  |  |  |  |  |
| --- | --- | --- | --- | --- | --- | --- | --- | --- | --- | --- | --- | --- | --- | --- | --- | --- |
|  |  | CEL |  |  |  |  |  |  |  |  |  |  |  |  |  |  |
|  |  | G-H1 | - 0.58 | 0.92 |  |  |  |  |  |  |  |  |  |  |  |  |
|  |  | MG-H1 |  |  | 0.49 | 0.66 |  |  |  |  |  |  |  |  |  |  |
|  |  | 3DG-H | - 0.49 | 0.90 |  | 0.89 | 0.54 |  |  |  |  |  |  |  |  |  |
|  |  | CMA | - 0.60 | 0.86 |  | 0.85 |  | 0.73 |  |  |  |  |  |  |  |  |
|  |  | PENT | 0.44 | - 0.52 |  |  |  |  | - 0.54 |  |  |  |  |  |  |  |
|  |  | GSP |  | 0.68 | 0.46 | 0.70 | 0.64 | 0.63 |  |  |  |  |  |  |  |  |
|  |  | AASA | - 0.61 | 0.71 |  | 0.62 |  | 0.57 | 0.78 | - 0.47 |  |  |  |  |  |  |
|  |  | GSA |  | 0.82 |  | 0.85 | 0.75 | 0.77 | 0.59 |  | 0.87 | 0.49 |  |  |  |  |
|  |  | DT |  | - 0.72 |  | - 0.66 |  | - 0.64 | - 0.72 |  |  | - 0.65 | - 0.51 |  |  |  |
|  |  | NFK |  |  |  |  |  |  | 0.48 |  |  |  |  |  |  |  |
|  |  | 3-NT |  |  |  |  |  |  |  |  | 0.53 |  |  |  |  |  |
|  |  | CP | - 0.51 | 0.88 | 0.45 | 0.90 | 0.51 | 0.79 | 0.73 |  | 0.81 | 0.60 | 0.89 | - 0.60 |  | 0.44 |
|  |  |  | FL | CML | CEL | G-H1 | MG-H1 | 3DG-H | CMA | PENT | GSP | AASA | GSA | DT | NFK | 3-NT |

Correlations statically significance (P<0.05) after a Bonferroni correction of 15 was applied.

**Table S4** Confusion matrix and nCorrect.

|  | | Algorithm 1 | | Algorithm 2 | | | |
| --- | --- | --- | --- | --- | --- | --- | --- |
|  | | Diseased | |  | Non-RA | eRA | eOA |
| nCorrect | | 42/48 | |  | 11/17 | 13/18 | 14/14 |
|  | | Predicted class | | Predicted class | | | |
|  |  | Disease | Control |  | Non-RA | eRA | eOA |
| Clinical class | Disease | 42 | 6 | Non-RA | 11 | 5 | 1 |
|  | Control | 1 | 18 | eRA | 5 | 13 | 0 |
|  | | | | oRA | 0 | 0 | 14 |

From 2-fold cross-validation. The values are representative of classification on 50% data as test set cross validation while the training was performed on the remaining 50% of the data.
